# Non-structural protein 1 of avian influenza A viruses differentially inhibit NF-κB promoter activation

**DOI:** 10.1186/1743-422X-8-383

**Published:** 2011-08-02

**Authors:** Muhammad Munir, Siamak Zohari, Mikael Berg

**Affiliations:** 1 Department of Biomedical Sciences and Veterinary Public Health, Swedish University of Agricultural Sciences (SLU), Ulls väg 2B, SE-751 89 Uppsala, Sweden; 2 Department of Virology, Immunobiology and Parasitology, National Veterinary Institute (SVA), Ulls väg 2B, SE-751 89 Uppsala, Sweden

**Keywords:** NS1 protein, avian influenza virus, NF-κB, allele A, allele B

## Abstract

**Background:**

Influenza virus infection activates NF-κB and is a general prerequisite for a productive influenza virus infection. On the other hand, non-structural protein 1 (NS1) suppresses this viral activated NF-κB, presumably to prevent expression of NF-κB mediated anti-viral response. NS1 proteins of influenza A viruses are divided into two groups, known as allele A and allele B. The possible functional relevance of this NS1 division to viral pathogenicity is lacking.

**Findings:**

The ability of NS1 protein from two avian influenza subtypes, H6N8 and H4N6, to inhibit NF-κB promoter activation was assessed. Further, efforts were made to characterize the genetic basis of this inhibition. We found that allele A NS1 proteins of H6N8 and H4N6 are significantly better in preventing dsRNA induced NF-κB promoter activation compared to allele B of corresponding subtypes, in a species independent manner. Furthermore, the ability to suppress NF-κB promoter activation was mapped to the effector domain while the RNA binding domain alone was unable to suppress this activation. Chimeric NS1 proteins containing either RNA binding domain of allele A and effector domain of allele B or vice versa, were equally potent in preventing NF-κB promoter activation compared to their wt. NS1 protein of allele A and B from both subtypes expressed efficiently as detected by Western blotting and predominantly localized in the nucleus in both A549 and MiLu cells as shown by *in situ *PLA.

**Conclusions:**

Here, we present another aspect of NS1 protein in inhibiting dsRNA induced NF-κB activation in an allele dependent manner. This suggests a possible correlation with the virus's pathogenic potential.

## Introduction

Within hours of host-pathogen interaction, the type 1 interferons (IFNs), an essential arm of innate immune response, are induced to initiate a range of antiviral processes. The binding of dsRNA, produced as a viral by-product (or administered externally such as poly I:C) to helicases or toll-like receptors (TLR), initiates a series of events culminating in the activation of two kinase complexes: TANK-binding kinase 1-inhibitor of kappa B-kinase ε (TBK1-IKK-ε) and IKK-α/β/γ [1]. TBK1-IKK-ε phosphorylates interferon regulatory factor 3 and 7 (IRF3 and IRF7) while IKK-α/β/γ phosphorylates and hence activates nuclear factor-κB (NF-κB) transcription factor. Activated NF-κB translocates to the nucleus where it induce the transcription of IFN-α and IFN-β as well as other pro-inflammatory cytokines together with ATF2/c-Jun (AP-1), p300 and CBP [2]. NF-κB consists of a family of transcription factors that play indispensable roles in mediating inflammation, immune responses to pathogen infection, proliferation, apoptosis, and other cellular activities [3]. Because of the essential role of NF-κB in stimulation of IFN-α/β synthesis, many viruses have evolved different strategies to subvert this system. The non-structural protein 1 (NS1) of influenza A viruses is one of best example having ability to prevent NF-κB activation.

It has been demonstrated that influenza virus infection activates the NF-κB and exhibit higher levels of replication in cells where NF-κB is pre-activated, suggesting that a NF-κB signalling pathway is a general prerequisite for a productive influenza virus infection [4]. Kumar *et al*., [5], made further clarifications that NF-κB signalling is intimately involved in the influenza vRNA synthesis. On the contrary, viral NF-κB activation is partially suppressed by the NS1 protein of influenza virus, presumably to prevent an overshooting expression of IFN-β. Thus, in the context of an influenza virus infection NF-κB appears to have a supportive function for viral replication that is dominant over its antiviral activity. The NS1 protein of influenza viruses consists of two domains: RNA binding domain (1-73 aa) and effector domain (74-230/237 aa). The N-terminal RNA binding domain is mainly responsible for interaction with RNA of several species whereas the C-terminal effector domain primarily mediates interactions with cellular proteins but also facilitates stabilization of the RNA binding domain [6].

Based on their amino acid sequences, NS1 proteins of influenza A viruses are divided into two groups, termed as allele A and allele B. There is little information available for the possible functional relevance of this difference to viral pathogenicity. However, recently we observed that allele A and B NS1s differ in their abilities to inhibit IFN-β promoter activation [7]. It has been demonstrated that H7N1 (A/FPV/Rostock/34), if carrying allele B of highly pathogenetic H5N1 (A/Goose/Guangdong/96), replicate more efficiently in human and mouse cell lines than wild-type H7N1 [8], which indicates that the NS1 protein is an essential determinant of influenza virus pathogenesis. The present study focuses on the abilities of allele A and B NS1 proteins to inhibit NF-κB promoter in cultured cells line.

## Materials and methods

The NS1 genes from four viruses belonging to different subtypes (H6N8 and H4N6), each containing both allele A and B were cloned in a mammalian expression vector pcDNA3.1+ (Invitrogen). These isolates were named H6N8-A (Allele A, A/mallard/Sw/412/05, EU518721) H6N8-B (Allele B, A/mallard/Sw/418/05, EU518722), H4N6-A (Allele A, A/mallard/Sw/818/05, EU518757) and H4N6-B (Allele B, A/mallard/Sw/795/05, EU518749). The chimeric NS1s were constructed as follow. The RNA binding domain (amino acids 1-73) of H6N8-A and effector domain (amino acids 74-230) of H6N8-B constitute H6N8 chiNS1 A/B and the RNA binding domain (amino acids 1-73) of H6N8-B and the effector domain (amino acids 74-230) of the H6N8-A constitute the H6N8 chiNS1 B/A. The RNA binding domains (H6N8-A-RNA and H6N8-B-RNA) and effector domains (H6N8-A-ED and H6N8-B-ED) of each allele were also cloned in pcDNA3.1+. The inserts in all the clones were confirmed by sequencing and expression ability was checked by TNT (TNT^® ^T7 Quick Coupled Transcription/Translation System, Promega) with ^35^S radiolabeling.

A549 cells, a type II alveolar epithelial cell line from human adenocarcinoma (ATCC, CCL 185), and mink lung cells (MiLu) were maintained as described [7]. The day before transfection, 24-well plates were seeded with ~2.5 × 10^4 ^cells per well. A 0.450 μg of each reporter plasmid (pNF-κB-Luc, containing an NF-κB promoter) and either NS1 expression plasmid or empty pcDNA3.1+ were transfected with FuGENE6 (Roche) at a ratio of 1:3 per well. Twenty-four hours post-transfection, the cultures were stimulated by adding 10 μg/ml poly I:C (dsRNA) (Invivogen). Twenty-four hours post-stimulation, the cells were lysed using ONE-Glo™ Luciferase Assay System (Promega) and the lysates were subjected to Wallac Victor^2^™ 1420 multilabel counter (Wallac Sverige AB) for luminescence. The plasmid pTA-Luc was transfected as a negative control for the estimation of background level of reporter gene activity and subtracted from all the values. The empty pcDNA3.1+ plasmid was transfected as a negative control and designated as mock treated. Each transfection experiment was repeated at least three times.

To check the expression of NS1 protein in the cells, *in situ *proximity ligation assay (PLA) was performed with the Duolink *in situ *PLA kit (Olink Biosciences). Western blotting was applied to check the total expression of NS1 protein, as we reported before [7].

## Results and discussion

The NS1 protein of influenza A viruses mediates resistance to the eukaryotic immune system for the efficient replication of viruses even in the presence of a functional anti-viral system. This property of NS1 has been well characterized in different studies [6,9]. In the present study, we expanded this notion by comparing the capacities of allele A and B NS1 proteins from two avian influenza subtypes to confer NF-κB promoter suppressive properties. Notably, these viruses were basically identical regarding other viral proteins except for the NS gene segment.

**Figure 1:**
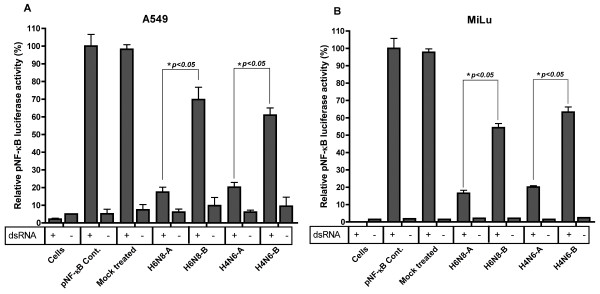
**Allele A and B NS1 proteins of avian influenza A viruses differentially block the dsRNA-induced expression of NF-κB promoter in A549 and MiLu cells**. (A) A549 cells were transiently co-transfected with NF-κB luciferase reporter plasmid, and vector encoding NS1 from H6N8-A, H6N8-B, H4N6-A, H4N6-B or empty pcDNA3.1+ vector (mock treated) or left un-transfected (cells). 24 hours post transfection, cells were stimulated with dsRNA (+) or left un-stimulated (-). 24 hours post induction, cell extracts were prepared and luciferase activity was measured using the ONE-Glo™ Luciferase Assay System (Promega) following the manufacturer's Instructions. The values were normalized and pNF-κB cont. was set to 100%. (B) MiLu cells were transfected as described for A549 cells. All the conditions were maintained for MiLu cells as that of (A). Error bars indicate standard deviations. The data shown are representative for three experiments with transfections performed in duplicate. * indicates a significant difference as determined by the student's t-test, with p-values of < 0.05.

In an initial experiment, 24 well plate was co-transfected with pNF-κB-Luc along with either NS1 expression plasmid for H6N8-A, H6N8-B, H4N6-A, H4N6-B or empty vector (mock treated) or left untreated (pNF-κB cont.). Relative luciferase activity was calculated 24 hours post-dsRNA stimulation. The A549 cells transfected with pNF-κB-luc and stimulated with dsRNA showed high activation of NF-κB promoter and was set to 100%. With the addition of NS1 from either of subtype or allele clearly diminished the NF-κB promoter activation (Figure 1A). Although, the level of inhibition of NF-κB promoter was evident for both alleles (A and B), surprisingly, it was significantly higher in allele A (~80%) compared to allele B (H6N8-B = 30% and H4N6-B = 40%) (p < 0.05). The non-stimulated set of same transfections showed non-specific background readings.

The results of the reporter assay proposed that NS1 proteins are involved in differential blocking of signal transduction pathways that lead to activation of the NF-κB promoters. Next, hypothesizing that these roles might be species-specific, we further examined this pathway in mink lung cells (MiLu). A corresponding picture appeared to that of A549 cells, where both allele A were consistently better in blocking the induction of NF-κB promoter compared to allele B NS1 protein of both subtypes. Allele B has shown an inhibition of 46% in H6N8 and 37% in H4N6 subtype of influenza virus and allele A has shown an inhibition of 84% and 80% in H6N8 and H4N6 compared to pNF-κB control, respectively (Figure 1B).

**Figure 2:**
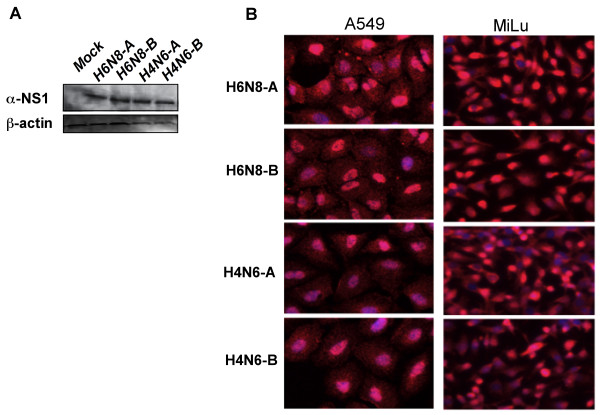
**Expression patterns of allele A and allele B NS1 proteins in subcellular compartments in A549 and MiLu cells**. (A) A549 cells were transfected with vector encoding NS1 from H6N8-A, H6N8-B, H4N6-A, H4N6-B or empty pcDNA3.1+ vector (mock treated). 24 hours post-transfection cells were lysed and subjected to Western blotting and probed with anti-NS1 and β-actin antibodies. (B) A549 and MiLu cells were transfected with vector encoding NS1 from H6N8-A, H6N8-B, H4N6-A, H4N6-B. 18 hours post transfection, cells were fixed and processed for *in situ *PLA, as recommended.

At this point, we were able to conclude that allele A is significantly more potent in inhibiting dsRNA induced NF-κB promoter than allele B NS1 proteins. However, we were interested to see whether this apparent limited inhibition by allele B NS1 might be due to an insufficient expression of NS1 protein leading to weaken its function. The whole A549 cell lysate was prepared after transfection of NS1 constructs from both alleles of H6N8 and H4N6, and subjected to Western blotting. As it is clear in Figure 2A, NS1 protein from all the isolates were expressed in high quantity and the level of allele A NS1 was comparable to NS1 protein of allele B. Nuclear localization of NS1 protein has been demonstrated as an essential character for the interaction of NS1 with the cleavage and polyadenylation specificity factor 30 (CPSF30) [10]. Furthermore, it has recently been highlighted that cellular expression pattern of NS1 protein is directly related to function of NS1 protein [11,12]. To pursue for these answers and to see the *in situ *expression of NS1 protein from both subtypes and both alleles, *in situ *PLA, a highly sensitive technique, was applied. The same experimental set-up was followed as that of Western blotting and instead of lysing the cells, 12 hours post-NS1 transfection cells were fixed and processed for PLA. As expected and consistent with Western blotting, a high expression of NS1 was observed for both subtypes and for both alleles. Moreover, at this time point, NS1 predominantly accumulated in the nucleus in both A549 cells and MiLu cells (Figure 2B). There was no detectable difference between alleles in terms of NS1 production as measured by the quantification of expressed NS1 proteins using BlobFinder software (data not shown). Thus, the results indicated that the low inhibition of NF-κB promoter in the presence of allele B NS1 protein was not due to a difference in allele B NS1 protein expression and accumulation in the cells.

**Figure 3:**
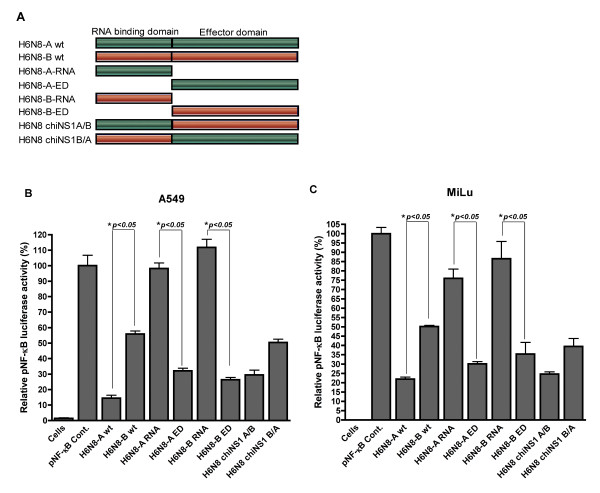
**Impact of RNA binding domain and effector domain of NS1 protein on dsRNA induced NF-κB promoter activity in A549 and MiLu cells**. (A) A schematic presentation for the construction of the RNA binding domain, the effector domain from both alleles of H6N8 and chimeric NS1s. (B) A549 cells were transiently co-transfected with NF-κB luciferase reporter plasmid, and vector encoding NS1 from H6N8-A, H6N8-B, H6N8-A-RNA, H6N8-A-ED, H6N8-B-RNA, H6N8-B-ED, H6N8 chiNS1 A/B, H6N8 chiNS1 B/A or empty pcDNA3.1+ vector (pNF-κB cont.) or left un-transfected (cells). 24 hours post transfection, cells were stimulated with dsRNA, cell extracts were prepared and luciferase activities were measured using the ONE-Glo™ Luciferase Assay System (Promega). The values were normalized and pNF-κB cont. was set to 100%. (C) All the conditions in MiLu cells transfection were maintained as practiced in (B). Error bars indicate standard deviations. The data shown are representative for three experiments with transfections performed in duplicate. * indicates a significant difference as determined by the student's t-test, with p-values of < 0.05.

Next, the genetic basis of inhibition of NF-κB promoter by NS1 from H6N8 allele A and B was investigated. For this purpose, the RNA binding domain and the effector domain from allele A and B of H6N8 (H6N8-A-RNA, H6N8-A-ED, H6N8-B-RNA and H6N8-B-ED) were constructed (a schematic diagram of these constructs is presented in Figure 3A). In a first round of experiments, NF-κB promoter was induced in A549 cells with dsRNA, in the presence of expression plasmids for RNA and effector domain of both allele A and B from H6N8. As shown in Figure 3B, the RNA binding domain from both allele A and B did not support NF-κB promoter inhibition and was non-significant when compared to positive control. On the contrary, the effector domain from both allele A and B led to the significant suppression of NF-κB promoter activation (~70%, when compared to pNF-κB cont.) (p < 0.05). In MiLu cells, a pattern corresponding to that of A549 cells was observed (Figure 3C) where the effector domain inhibit NF-κB promoter whereas the RNA binding domain lacks this ability. These results indicated that the ability to block dsRNA induced NF-κB signaling is mapped into the effector domain and that the RNA binding domain alone is unable to block NF-κB promoter activity in both A549 and MiLu cells. Previous studies have indicated that the effector domain of NS1 protein is mainly involved in regulation of cellular activities by protein-protein interaction but is also essential for the dimerization and stability of RNA domain [6,10,13-15]. Consistent with these results, it is also likely that the lack of effector domain in NS1 protein influences the three-dimensional structure and dimerization of the RNA domain, which affects the function in the suppression of NF-κB promoter activation.

To further prove this notion, two chimeric NS1s were constructed containing either RNA binding domain of allele A and effector domain of allele B (H6N8 chiNS1 A/B) and vice versa (H6N8 chiNS1 B/A). Cell transfection was performed in A549 cells as outlined before with the difference that chimeric NS1 from both allele A and B were used. Interestingly, NF-κB promoter inhibition was reversed in both chimeric NS1 and was comparable to their wild type (wt) (Figure 3B). This ability of chimeric NS1 was reproducible in MiLu cells and was non-significant when compared to corresponding wt NS1 protein. Taken together, these data demonstrated that the two domains are probably functionally interactive in a co-operative manner to result in their overall NF-κB promoter inhibition. It is also to observe that within RNA binding domain, a unique motif (^21^RFADQELG^28 ^in alleles A and ^21^LLSMRDMC^28 ^in allele B) may have biological relevance, which warrant further investigation. Moreover, allele A NS1 proteins differ with 71 aa (~31%) from allele B NS1 protein of both H6N8 and H4N6 subtypes. These amino acid differences most likely reflect differences in the respective protein function and would be interesting to study in terms of the biological significance in the context of viral infections.

## Conclusions

In conclusion, the results of this study demonstrated that NS1 proteins from avian influenza A viruses (H6N8 and H4N6 subtypes) have the ability to inhibit NF-κB promoter activation and suggest a possible correlation with the virus's pathogenic potential. Mapping of the NS1 protein domains involved in inhibition of NF-κB promoter showed that NS1 devoid of RNA binding domain still exhibited property of NF-κB promoter inhibition. Conversely, RNA binding domain alone loses this inhibitory activity. However, for full elucidation, further experiments involving fusion of dimerization domain to that of RNA binding domain are required. Moreover, the full length NS1 protein is functionally interactive and can efficiently block NF-κB promoter activation regardless of RNA binding domain (either of allele A or allele B). Apart from NF-κB signaling, allele A and B NS1 protein from both subtypes were expressed predominantly in nucleus but also in cytoplasm in transfected human A549 and MiLu cells. On the basis of this work, we presented another dimension of NS1 protein in inhibiting dsRNA induced NF-κB activation that this character of NS1 is allele dependent.

## Competing interests

The authors declare that they have no competing interests.

## Authors' contributions

Conceived and designed the experiments: MM, SZ, MB. Performed the experiments: MM, SZ. Analyzed the data: MM, SZ, MB. Wrote the paper: MM, SZ, MB. All the authors have read and approved the final manuscript.
